# Heavy Metal Contamination in Edible Species from Quintero-Puchuncaví Bay: Risks Associated with the Icon Industrial Complex in Central Chile

**DOI:** 10.3390/toxics14050397

**Published:** 2026-05-06

**Authors:** Stephanny Curaz-Leiva, María José Díaz, Iván Sola, Jhoel Ruiz, Macarena Pérez, Daniel González-Labra, Brittany Paredes-Ocaranza, M. Gabriela Lobos, Celine Lavergne, Sebastián A. Klarian, Verónica Molina, Claudio A. Sáez

**Affiliations:** 1 Programa de Doctorado Interdisciplinario en Ciencias Ambientales, Facultad de Ciencias Naturales y Exactas, Universidad de Playa Ancha, Valparaíso 2340000, Chile; stephanny.curaz@alumnos.upla.cl (S.C.-L.); macarenaperez@alumnos.upla.cl (M.P.); d_gonzalez@alumnos.upla.cl (D.G.-L.); brittany.paredes@alumnos.upla.cl (B.P.-O.); 2 Multidisciplinary Institute for Environmental Studies “Ramon Margalef” (IMEM), University of Alicante, 03080 Alicante, Spain; 3 Departamento de Ciencias del Mar y Biología Aplicada, Facultad de Ciencias, Universidad de Alicante, 03690 Alicante, Spain; 4 HUB AMBIENTAL UPLA, Universidad de Playa Ancha, Valparaíso 2340000, Chile; mariajose.diaz@pucv.cl (M.J.D.); ivan.sola@uc.cl (I.S.); lavergne@icm.csic.es (C.L.); 5 Departamento de Ciencias y Geografía, Facultad de Ciencias Naturales y Exactas, Universidad de Playa Ancha, Valparaíso 2340000, Chile; 6 Centro de Investigación Oceanográfica COPAS COASTAL, Universidad de Concepción, Concepión 4030000, Chile; 7 Escuela de Ciencias del Mar, Facultad de Ciencias del Mar y Geografía, Pontificia Universidad Católica de Valparaíso, Valparaíso 2362807, Chile; 8 Departamento de Ingeniería Hidráulica y Ambiental, Pontificia Universidad Católica de Chile, Santiago 8320000, Chile; 9 Instituto para el Desarrollo Sustentable, Pontificia Universidad Católica de Chile, Santiago 8320000, Chile; 10 Laboratorio de Química Ambiental, Instituto de Química y Bioquímica, Facultad de Ciencias, Universidad de Valparaíso, Valparaíso 2340000, Chile; jhoel.ruiz.pinilla.alexander@gmail.com (J.R.); gabriela.lobos@uv.cl (M.G.L.); 11 Departament de Biología Marina i Oceanografia, Institut de Ciències del Mar, Consejo Superior de Investigaciones Científicas, 08003 Barcelona, Spain; 12 Centro de Investigación Marina Quintay CIMARQ, Facultad de Ciencias de la Vida Universidad Andrés Bello, Viña del Mar 2520000, Chile; sebastian.klarian@unab.cl; 13 Department of Ecology and Evolutionary Biology, University of Connecticut, Storrs, CT 06269, USA

**Keywords:** bioaccumulation, biomagnification, marine pollution, metals

## Abstract

Although Quintero-Puchuncaví Bay, Chile, is a coastal area historically known to be subject to multiple industrial pressures, few studies have focused on the associated risks to marine ecosystems and, through edible species, to human health. We studied concentrations of Cd, Cr, Cu, Mn, Pb, V, Zn, and Hg in marine species and sediments from Quintero-Puchuncaví Bay and a reference site. Results were compared with seafood safety guidelines, and target hazard quotients (THQs) were evaluated. Sediments and biota from the impacted area generally exhibited higher metal concentrations. Zinc (Zn) and copper (Cu) levels were the highest across all species, particularly in crustaceans, reflecting both physiological requirements and anthropogenic inputs. Cadmium (Cd) concentrations were higher in pelagic species from the impacted bay, but no differences were found in sediments or benthic species, suggesting the influence of upwelling conditions. Comparison with seafood safety guidelines revealed that Cd and Pb concentrations exceeded permissible limits in crabs, fish, and mussel species, and THQ ≥ 1 values were found for Cd concentrations in benthic species from the impacted bay, highlighting potential risks to consumers. The absence of permissible thresholds for certain environmentally relevant metals in Chilean regulations underscores the need to align with international standards, certainly to protect coastal ecosystems and human health.

## 1. Introduction

Metals occur naturally in the marine environment at low background concentrations. However, anthropogenic factors, including port operations, urbanization, and industrialization, have elevated heavy metal levels in coastal ecosystems [1,2]. Essential metals, such as copper (Cu), manganese (Mn), vanadium (V), and zinc (Zn), facilitate biological functions at trace levels but can induce toxicity at higher concentrations. For instance, Cu serves as a cofactor for enzymes, such as copper nitrite reductase, which mediates microbial biogeochemical processes, such as the nitrogen cycle [3]. Conversely, elevated Cu exposure (>2.5 µg/L) impairs larval settlement in the scallop *Argopecten purpuratus* by altering the expression of genes associated with stress, development, and differentiation [4]. Even though these metals are essential, it is clear that beyond certain threshold concentrations they can pose a risk to the biology of organisms.

Non-essential heavy metals, such as cadmium (Cd), lead (Pb), and mercury (Hg), have no known biological roles and are toxic even at trace concentrations [5,6]. Mechanistically, Cd obstructs Ca transport within proteins, inducing hypocalcemia, while Pb mimics Ca during gill uptake, increasing nitrogen catabolism [7]. Similarly, Hg triggers cellular alterations in adult fish and causes developmental malformations in larvae [8]. Quantifying these elements in marine biota is therefore essential for assessing ecological status and evaluating mid-to-long-term toxicological impacts.

Once introduced into the environment, metals may bioaccumulate, a process in which internal concentrations increase because intake through dietary absorption or surface transport exceeds excretion rates [9]. This process is influenced by feeding strategies, exposure duration, and environmental conditions [10]. For example, in Laizhou Bay, China, bivalves such as *Scapharca subcrenata*, *Mactra veneriformis*, and *Ruditapes philippinarum* exhibited significant inter-specific differences in Cd, Hg, and Zn accumulation, likely linked to tissue-specific physiological functions [11]. Similarly, in northern Egypt, *Mugil cephalus* showed higher metal accumulation in the liver than *Pagrus pagrus* and *Sardinella aruita*, a pattern associated with its detritivorous feeding behavior near contaminated sediments [12]. Thus, the inclusion of other abiotic matrices, such as sediments, complements and improves environmental diagnosis.

Excessive metal accumulation induces physiological disturbances that alter growth and reproduction in marine invertebrates and fish [13,14]. Furthermore, certain metals undergo biomagnification, increasing in concentration at higher trophic levels [5]. Consequently, top predators are highly vulnerable to toxic effects, which may subsequently pose health risks to the human population through seafood consumption. The general population is commonly exposed to Cd and Hg through the ingestion of contaminated food [15]. Chronic exposure to Cd and Hg via contaminated fish and shellfish is associated with neurological impairments, organ damage, and carcinogenesis [16]. Therefore, monitoring coastal metal concentrations is essential to inform conservation measures and human health contingencies.

In heavily impacted regions, heavy metals are often discharged directly into coastal waters via untreated sewage and industrial effluents. Notable examples include the industrial hub of Chattogram, Bangladesh [17], and the Pearl River Estuary in China, where four decades of manufacturing have made metal pollution a primary environmental concern [18], highlighting the urgent need to evaluate environmental contamination in regions subjected to long-term industrial pressures. The Quintero–Puchuncaví Bay in central Chile represents a similar case of historic anthropogenic impact. Located in a shallow horseshoe-shaped bay open to the north (approx. 40 m depth), this area has been a major industrial center since the 1950s. After 1991, with the implementation of initial environmental regulations, scientific studies began to demonstrate the extent of the anthropogenic impact [19]. Today, the “Ventanas Industrial Complex” encompasses more than ten facilities—including copper refineries, coal-fired power plants, and chemical facilities—that serve as significant sources of metal pollution [20].

The consequences of this situation are well documented. High levels of atmospheric pollution have led to air quality levels harmful to human health and episodes of acid rain; these have also been shown to transport metals into marine waters [21]. In 1993, the Chilean government declared the area “saturated.” Since then, elevated metal concentrations in marine resources have forced reductions in fishing quotas and commercial restrictions, affecting local artisanal fisheries [22]. Multiple studies confirm heavy metal enrichment in abiotic matrices; sediments show higher concentrations of Al, Fe, Cu, Mn, and Pb compared to reference sites such as Faro Curaumilla [23], and elevated levels of Cu, Zn, Pb, and As have been associated with copper smelting activities [24]. Another study reported the presence of Cu, Pb, Cd, and Mo, confirming the enrichment of Pb and Mo in marine sediments [25]. In seawater, Cu and As frequently exceed US EPA standards in the “National Recommended Water Quality Criteria” [26]. Biological assessments have also detected Cu, Cd, and Zn in bivalves (*Perumytilus purpuratus*, *Semelle solida*, and *Tagelus dombeii*), which exhibited higher Cu concentrations compared to other anthropogenically impacted areas, such as Antofagasta and Chañaral [27], and in gastropods (*Concholepas concholepas*), with levels often surpassing safety thresholds established by the Chilean (SD. 977/96) [28] and European Union regulations [29]. Therefore, there is a history of metal contamination known to have an impact on metal levels in marine organisms in the area. It is important to maintain an accurate, up-to-date assessment not only for the purposes of marine environmental assessment but also considering that many organisms in the area are harvested for human consumption, potentially posing a public health risk.

Updated studies focusing on edible species are crucial for addressing imminent threats to both the ecosystem and public health, especially in areas such as Quintero-Puchuncaví Bay, which is currently classified as a zone of persistent ecological risk. Despite this, previous studies have primarily focused on individual species. The main objective of this study was to assess heavy metal concentrations in various edible marine species considered fishery resources in the Quintero-Puchuncaví area. To this end, we conducted the most comprehensive study to date, involving the collection and analysis of species commonly consumed by the local population, gathered from both industrially impacted and control sites. The results were also discussed within the framework of national and international regulations regarding recommended metal levels in food products and potential consequences for human health.

## 2. Materials and Methods

### 2.1. Sample Collection

**Figure 1:**
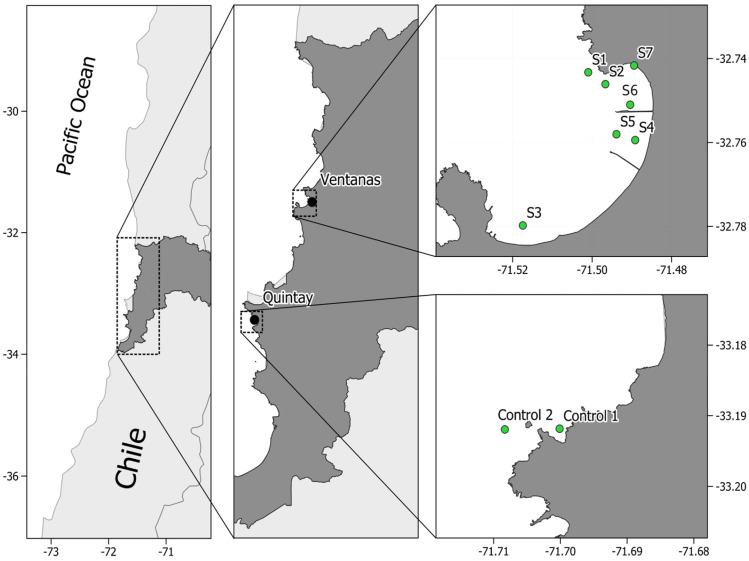
Map of the Valparaíso region, showing sampling sites along Quintero–Puchuncaví Bay, referred to as “Ventanas” after the name of the industrial complex (**upper**), and Quintay Bay (**lower**). Sites S1, S2, S3, S4, S5, S6, and S7: Quintero–Puchuncaví Bay. Control 1 and Control 2: Quintay Bay.

Subtidal organisms were collected from six sites, and a single coastal night sampling was conducted in the industrially impacted area during spring 2022 (Table S1, Figure 1). Sites were selected based on known marine circulation patterns for water, sediments, and associated metals, as well as their proximity to major industrial discharge points [30]; therefore, most stations were located in the northern portion of the bay. For comparison, a control location, the Quintay Bay area, also located in the Valparaíso province, was selected (Figure 1). This bay is a site with no history of metal pollution [31].

All biological specimens were collected by diving or coastal sampling using new plastic hand nets to prevent trace metal contamination. Based on their presence and representative biomass, ten species were collected from Quintero–Puchuncaví Bay and eight from Quintay. In both areas, the fish species *Pinguipes chilensis*, *Aplodactylus punctatus*, and *Cheilodactylus variegatus* were sampled, along with the mollusks *Concholepas concholepas* and *Fissurella* spp. and the crustaceans *Romaleon setosum* and *Homalaspis plana*. The only macroalga collected was the brown seaweed *Lessonia trabeculata*. In Quintero–Puchuncaví Bay, two additional species were included: the mollusk *Argopecten purpuratus* and the crab *Ovalipes trimaculatus*. At each site, three independent sediment samples were collected using plastic equipment.

Samples were stored in coolers and immediately transported to the laboratory. Sediments were kept at −80 °C in acid-cleaned (10% _HNO3_) plastic containers until analysis. Organisms were washed and frozen at −20 °C following Lobos et al. (2019) [32]. Seaweed fronds were used, and only the edible muscle tissue was excised from animal specimens. For scallops and limpets, we generated composite samples (triplicate pools) to ensure sufficient biomass for analysis.

### 2.2. Metal Analysis

Samples were analyzed for Cd, Cr, Cu, Mn, Pb, V, Zn, and Hg. Tissues and sediments were freeze-dried to constant weight at −50 °C for 95 h. Biological samples were pulverized, while sediments were homogenized using an agate mortar, and particle size was standardized using a sieve (62 µm) [23].

For Hg determination, 0.08 g of biological tissue or 0.10–0.15 g of sediment was analyzed using a direct mercury analyzer (model DMA-80, Milestone, Sorisole, Italy) [33]. For Cd, Cr, Cu, Mn, Pb, V, and Zn analysis, the EPA Method 3052 protocol was adapted in accordance with the manufacturer’s instructions. Briefly, 0.5 g of dry sample was digested with 10 mL of HNO_3_ (65%) in a Teflon vial using microwave digestion (model MWD-700, Metash, Shanghái, China). The resulting transparent digest was diluted to 100 mL with ultrapure water. Metal concentrations were determined by Inductively Coupled Plasma Optical Emission Spectrometry (ICP-OES; model iCAP Pro, Thermo Scientific, Waltham, MA, USA) [34]. The instrument operated in an Axial iFR view mode, argon gas flow, 15/min plasma and 0.2/min auxiliary flow. The following wavelengths were used: Cd (228.802), Cr (283.563), Cu (324.754), Mn (257.610), Pb (220.353), V (309.311), Zn (213.856), and Hg (253.7). Method detection limits (MDLs) post digestion were 0.2 µg/L for Cd, Cr, Cu, Mn, Pb, V and Zn and 1 ng/L for Hg. For calibration curves, the linear working range was 5–200 µg/L, with R^2^ ≥ 0.9998. To ensure precision and accuracy, the methodology was also applied to certified reference materials (BCR-279, ERM-CE278K, DORM-4, ERM-CE464, and BCR-710; Health, Consumers and Reference Materials, European Commision, Geel, Belgium). After every ten samples, reference materials and blanks were applied. The standard addition method was used to verify that there was no matrix effect. Recovery rates ranged from approximately 90% to 110%, and the reference standard deviation (SD) was <8%.

### 2.3. Health Risk Assessment and Target Hazard Quotient (THQ)

To compare our results with previous studies and with national and international guidelines, all metal concentrations were converted to wet weight using a moisture conversion factor of 0.25. This factor falls within the commonly accepted moisture range for marine organisms [35] and is consistent with established values for Chilean seafood [36].

Since the non-essential heavy metals Cd, Pb, and Hg are frequently considered in seafood safety guidelines, they were selected to estimate the Target Hazard Quotient (THQ). The THQ assesses the non-carcinogenic risk associated with seafood consumption in relation to their oral reference doses, according to the equation:

$$THQ=\;\frac{EF\;\times \;ED\;\times \;FIR\;\times \;C}{RfD\;\times \;BW\;\times \;AT}\;\times \;10^{-3}\;\;$$

where *EF* corresponds to the exposure frequency (365 days/year), *ED* is the exposure duration (70 years), *FIR* is the ingestion rate (g/day), *C* is the mean concentration of each metal (wet weight), *RfD* values indicate the oral reference doses (mg/kg/day) (Cd = 0.001, Pb = 0.004, and Hg = 0.0001) [37], *BW* is the average body weight (kg), and *AT* is the averaging time for non-carcinogenic effects (*EF* × *ED*) (365 days/year × 70 years). According to Vergara et al. [38], the average ingestion rate (*FIR* = 39.45 g/day) was estimated from the National Oceanic and Atmospheric Administration [39], and the average body weight for women (75.5 kg) and men (85.1 kg) was obtained from the Eglitis dataset [40]. If *THQ* < 1, it indicates no health risk to consumers; if *THQ* ≥ 1, it indicates a possible non-carcinogenic health risk.

### 2.4. Statistical Analysis

Statistical analyses were restricted to a subset of species with sufficient replication: *P. chilensis*, *A. punctatus*, *C. concholepas*, *H. plana*, and *R. setosum*. The significance level was set at *p* < 0.05, and data were tested for normality (Shapiro–Wilk test) and homogeneity of variance (Levene’s test). When assumptions were met, a *t*-test was applied; otherwise, non-parametric tests were performed using the statistical software R (V.4.3.1). Sediment metal concentrations were compared using the same statistical approach. Additionally, a Principal Component Analysis (PCA) was performed on log-transformed data to evaluate metal relationships across species. A Redundancy Analysis (RDA) was utilized to identify the influence of feeding habits on metal profiles.

## 3. Results

### 3.1. Metal Bioaccumulation

**Figure 2:**
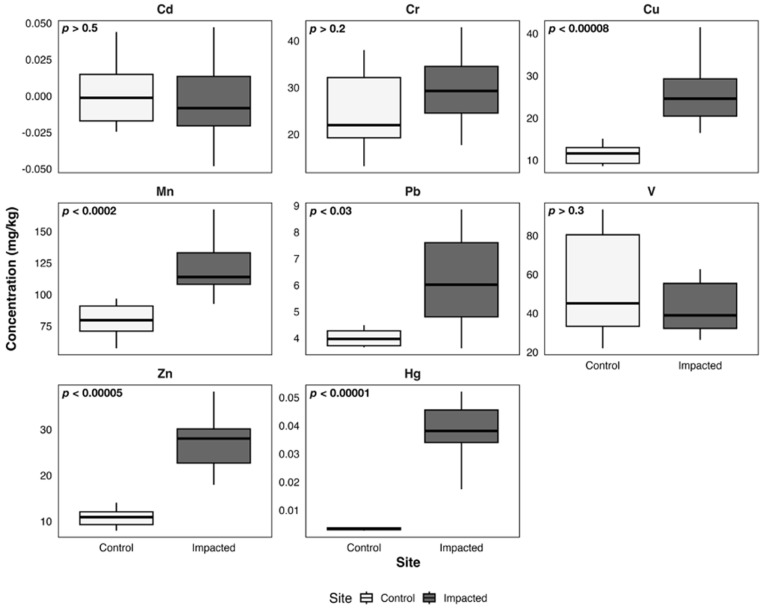
Boxplots of metal concentrations (mg/kg) in sediments from the control and impacted areas of Quintero-Puchuncaví Bay. Boxplots represent the median (central line) and the interquartile range (25–75%). Statistical comparisons are shown in each panel.

Sediment heavy metal concentrations in the control (Quintay) and impacted (Quintero–Puchuncaví) areas are presented in Figure 2; to display clearer trends with stronger statistical significance, results from the sites were grouped by control and impacted area. No significant differences were identified for Cd, Cr, and V between areas (*p* > 0.05), whereas all other metals exhibited statistically significant inter-site variation (*p* < 0.05) with higher concentrations in the impacted area.

Sample sizes and mean concentrations for all analyzed elements (Cd, Cr, Cu, Mn, Pb, V, Zn, and Hg) across species are summarized in Table 1. Regardless of the sampling area, Zn reached the highest concentrations across all taxa, with crustaceans exhibiting the maximum values (179.23 ± 23.30 to 240.40 ± 31.25 mg/kg). In contrast, Cd displayed the lowest concentrations among fish species in both areas (0.04 ± 0.007 to 0.19 ± 0.03 mg/kg). For mollusks, crustaceans, and the primary producer, Hg remained the least abundant element, and the lowest concentrations were found in mollusks; however, fish species exhibited the highest concentration in both the control (0.02 ± 0.000022 to 0.51 ± 0.009 mg/kg) and the impacted (0.32 ± 0.001 to 0.48 ± 0.006 mg/kg) areas.

**Figure 3:**
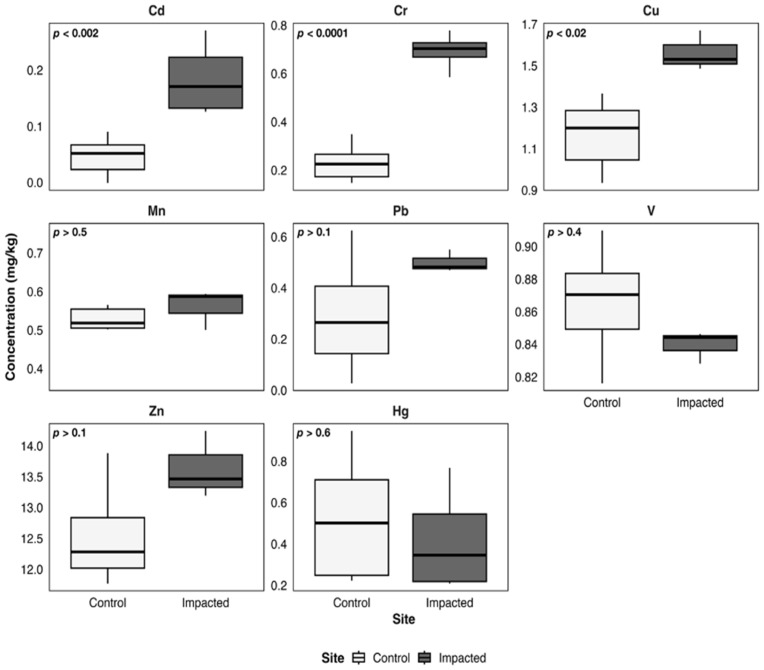
Boxplots of metal concentrations (mg/kg) in *P. chilensis* from the control and the impacted area of Quintero-Puchuncaví Bay. Boxplots represent the median (central line) and the interquartile range (25–75%). Statistical comparisons are shown in each panel and are considered significant at the 95% confidence level.

**Figure 4:**
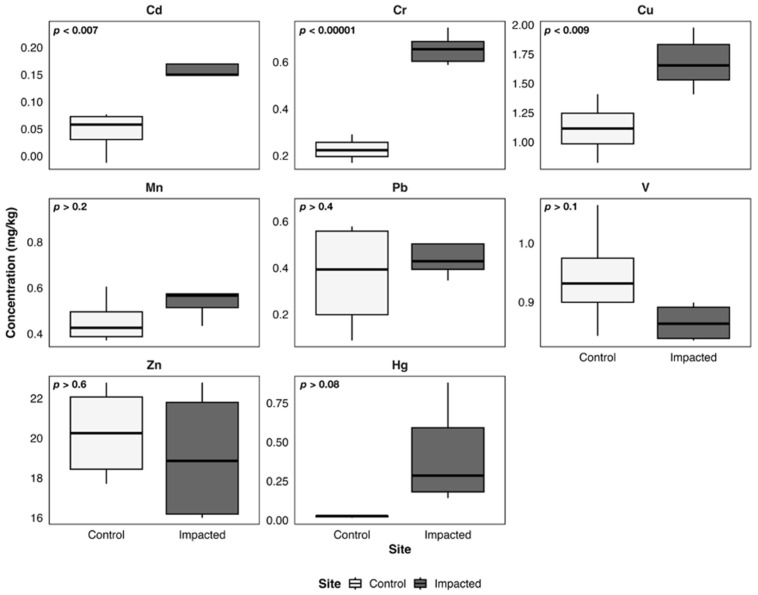
Boxplots of metal concentrations (mg/kg) in A. punctatus from the control and the impacted area of Quintero-Puchuncaví Bay. Boxplots represent the median (central line) and the interquartile range (25–75%). Statistical comparisons are shown in each panel and are considered significant at the 95% confidence level.

**Figure 5:**
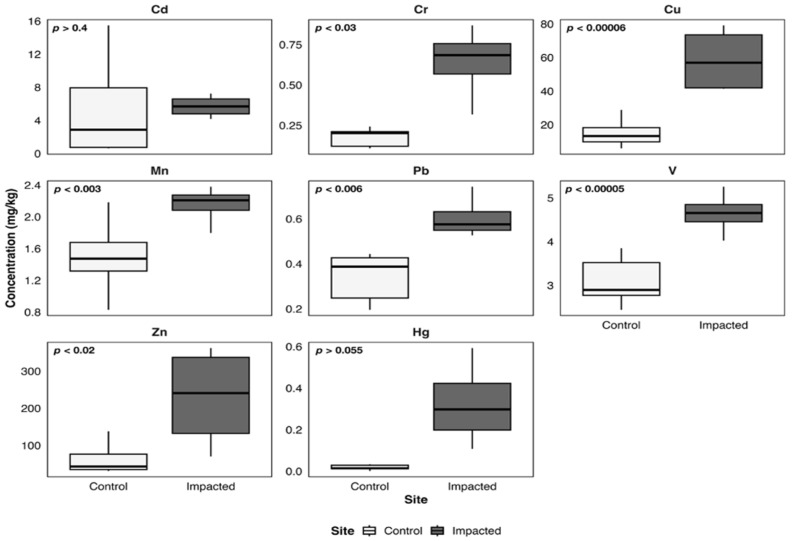
Boxplots of metal concentrations (mg/kg) in *C. concholepas* from the control and the impacted area of Quintero-Puchuncaví Bay. Boxplots represent the median (central line) and the interquartile range (25–75%). Statistical comparisons are shown in each panel and are considered significant at the 95% confidence level.

**Figure 6:**
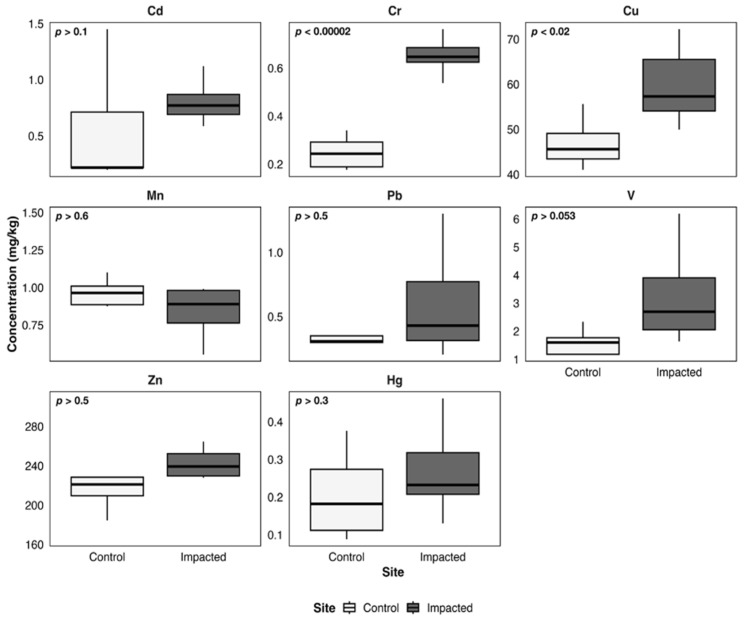
Boxplots of metal concentrations (mg/kg) in *H. plana* from the control and the impacted area of Quintero-Puchuncaví Bay. Boxplots represent the median (central line) and the interquartile range (25–75%). Statistical comparisons are shown in each panel and are considered significant at the 95% confidence interval.

**Figure 7:**
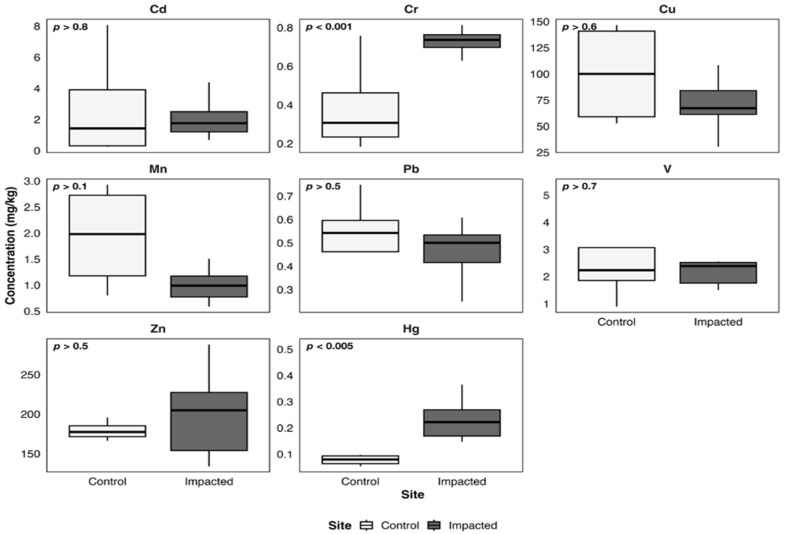
Boxplots of metal concentrations (mg/kg) in *R. setosum* from the control and the impacted area of Quintero-Puchuncaví Bay. Boxplots represent the median (central line) and the interquartile range (25–75%). Statistical comparisons are shown in each panel and are considered significant at the 95% confidence level.

Figure 3, Figure 4, Figure 5, Figure 6 and Figure 7 illustrate metal concentrations in statistically evaluated species. In the fish *P. chilensis* (Figure 3) and *A. punctatus* (Figure 4), significant differences between sites were found for Cd (*p* < 0.002 and *p* < 0.007), Cr (*p* < 0.0001 and *p* < 0.00001), and Cu (*p* < 0.02 and *p* < 0.009). The gastropod *C. concholepas* exhibited significant variation in nearly all analyzed metals, including Cr, Cu, Mn, Pb, V, and Zn (Figure 5). In crustaceans, H. plana showed significant differences in Cr and Cu (Figure 6), while *R. setosum* differed in Cr and Hg (Figure 7). Overall, results indicate a consistent trend toward higher metal concentrations in the impacted area.

Supplementary Figures S1–S3 present data for the remaining species. Although limited sample sizes precluded statistical testing for some species, the trends are clear: almost all metal concentrations were elevated in the Quintero–Puchuncaví area. The primary producer *L. trabeculata* followed this pattern for all metals except Cd (Figure S3), a trend also observed in the benthic crustacean *R. setosum*.

### 3.2. Principal Component Analysis

The PCA results indicate that the first two principal components (PCs) explained 64.86% of the total variance in the control area (Figure S4A) and 68.48% in the impacted area (Figure S4B). In both areas, PC1 was mainly driven by Zn, Cu, Cd, Mn, and V, whereas PC2 was driven by Pb and Cr, and fish species clustered along the negative axis of PC1 in association with Hg.

Based on feeding habits, in the control area, PC1 distinguished two groups on the positive side of the axis: (1) the benthic carnivorous species *C. concholepas, R. setosum,* and *H. plana*, associated with Cu and Zn, and (2) *Fissurella* spp. and *L. trabeculata*, associated with Pb. In the impacted area, *O. trimaculatus* grouped together with the benthic carnivores, while *A. purpuratus* clustered with *Fissurella* spp. and *L. trabeculata*.

### 3.3. Health Risk Assessment

**Figure 8:**
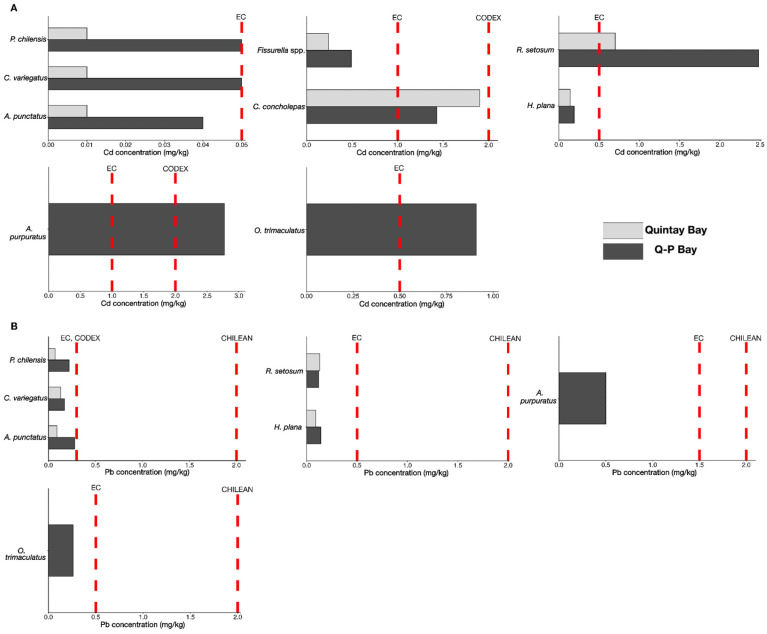
Evaluation of metal contamination in fish, mollusk, and crustacean species (mg/kg w.w.) determined in our investigation, compared with different regulatory guidelines. The graphs focus on the most relevant metals observed in species regarding their concentrations and potential risks for human health: (**A**) Cd concentration and safety thresholds for species above or close to any threshold limit and (**B**) Pb concentration and safety thresholds for species above or close to any threshold limit. EC: European Commission Regulation 2023/915 [41]. CODEX: CODEX Alimentarius [42]. CHILEAN: Chilean Supreme Decree No. 977 [28].

When comparing the Chilean Supreme Decree on Food Safety Regulations (SD. 977/96) [28] with international guidelines, results revealed that several species exceeded safety thresholds for Cd or Pb: *P. chilensis*, *A. punctatus*, *C. variegatus*, *R. setosum*, *O. trimaculatus*, and *A. purpuratus* from the impacted area and *R. setosum* from the control area (Figure 8, Table S2). Due to the lack of threshold limits for Cd in gastropod species, threshold limits set for bivalves were used in *Fissurella* spp. and *C. concholepas*.

The THQ values are in Table S3. In Q-P Bay, Cd concentrations in *R. setosum* and *A. purpuratus* showed THQ ≥ 1 values for both women and men, while Cd concentrations displayed THQ values close to 1 for *C. concholepas* in Quintay Bay. Pb and Hg concentrations presented THQ < 1 for all studied species.

## 4. Discussion

### Heavy Metal Bioaccumulation

Heavy metal concentrations in species from Quintero–Puchuncaví Bay generally exceeded those from Quintay. In both areas, Zn and Cu exhibited the highest concentrations across fish, mollusks, and crustaceans. This pattern aligns with global findings and suggests that these levels are driven by both environmental proximity to pollution and the essential metabolic roles of these elements [43]. These findings corroborate historical data and maintain the area at the center of ecotoxicological attention. Zinc (Zn) was previously identified as the dominant metal in species such as *P. purpuratus*, *S. solida*, and *T. dombeii* [27], underscoring the long-term persistence of trace metal enrichment. It is important to mention that due to the absence of certain species in sampled locations, the number of collected individuals may not be sufficient for statistically significant representation. However, they were still collected and analyzed given their ecological and human edibility relevance and because, even though the data for some of them were low, the trends were relevant for an up-to-date baseline of metal status for these organisms. While further efforts should be made to study a larger number of individuals per species, the sample sizes achieved in this investigation nonetheless may serve as an indirect demonstration of the low richness and abundance of organisms in Quintero-Puchuncaví Bay.

Crustaceans exhibited the highest zinc levels in both study areas, with a clear trend toward higher levels in Quintero-Puchuncaví Bay. Sediment analysis supported these observations; previous research has linked high sediment zinc levels to smelting and power plant emissions [24]. However, no differences were detected in zinc concentrations between the crab species in the two areas. Notably, Zn concentrations in *R. setosum* (179.23 ± 17.39 mg/kg in Quintay and 201.54 ± 19.69 mg/kg in Quintero–Puchuncaví) exceeded those reported for other contaminated sites such as Caldera Bay (103.5 mg/kg) [44] or San Jorge Bay (91.0 ± 10.7 mg/kg) [45], both of which are well known for metal contamination associated with mining activities. Similar patterns have been described worldwide. For instance, in *Austruca iranica* from a port-impacted area (Pakistan), Zn showed the second-highest accumulation after Fe, ranging from 63.38 mg/kg to 106.63 mg/kg [46], and in a bay influenced by salmon aquaculture in the Orkney Islands (Scotland), Zn concentrations in *Necora puber* were higher than those of Cu but lower than those of essential macroelements such as Ca and Mg [47].

The elevated Zn concentrations at Quintay—our control site—may be related to its physiological role, as Zn is a cofactor for approximately 200 enzymes and is essential for hemocyanin synthesis and metallothionein regulation [48,49]. Thus, the observed Zn levels may reflect internal homeostatic regulation and storage rather than exclusively anthropogenic forcing. Similar phenomena have been documented in protected areas elsewhere, such as Essaouira (Morocco), where high Zn concentrations in *Mytilus galloprovincialis* ranged from 123.47 mg/kg to 342.73 mg/kg and were attributed mainly to natural geochemical background and terrigenous inputs rather than to anthropogenic sources, unlike Cd, Cr, and Pb [50,51].

Cu exhibited the next highest concentrations, with differences observed across nearly all species. In Quintero-Puchuncaví Bay, substantial Cu loads enter the bay via atmospheric transport, industrial and domestic effluents, and port activities; as a result, marine sediments act as both reservoirs and secondary sources of pollutants in the water column [22]. Consequently, marine species show elevated Cu in their tissues. For instance, the macroalga *Macrocystis pyrifera* and the sea urchin *Tetrapygus niger* from Caleta Horcón, located 5 km north of Quintero-Puchuncaví Bay, exhibited higher Cu levels than individuals from Algarrobo Beach, along with increased concentrations of other pollutants such as Cd, As, and naphthalene [52]. In our study, the highest Cu levels were observed in the crab *O. trimaculatus* (108.73 ± 6.1 mg/kg), which inhabits sandy bottoms where sediment-bound metals can be mobilized via dietary absorption or transport across permeable surfaces such as gills and the exoskeleton [44,53].

Metal intake can result from both endogenous and external factors, such as geographical location or diet [54]. For example, *C. concholepas*, a carnivorous intertidal gastropod, may accumulate high metal concentrations due to its efficient assimilation and lower efflux rates [55]; however, in our study, it exhibited lower Cu concentrations than *Fissurella* spp. in the Quintero–Puchuncaví. This difference may reflect distinct feeding habits, as *Fissurella* spp. are omnivores with a preference for algae [56], and in our study the kelp *L. trabeculata* presented higher Cu levels in the impacted bay, consistent with metal intake via food consumption and previous findings in the area [57], since metal concentrations in primary producers are proportional to levels in the surrounding water [58].

Interestingly, *R. setosum* deviated from general trends, showing higher Cu concentrations in Quintay Bay. Central Chile is characterized by Eastern Boundary Upwelling Systems (EBUS), the most productive region in the world’s oceans, which creates high temporal variability that regulates the oceanographic environment [59]. Coastal upwelling at Punta Curaumilla—adjacent to Quintay—brings nutrient-rich, trace-metal-enriched waters to the surface [60]. Trace elements taken up by planktonic species are transferred and deposited via direct sedimentation or sinking fecal pellets, enriching deeper waters and sediments and being recycled through biogeochemical cycles [61,62]. This natural enrichment explains why benthic species and the kelp *L. trabeculata* also showed elevated Cd levels in Quintay. In EBUS regions, Cd is often enriched in sediments because it functions as a micronutrient for plankton and is rapidly sequestered in highly productive waters [23], as documented in other areas, such as the California, Namibian, and Peruvian upwelling systems [63,64]. Thus, Cu and Cd levels in Quintay likely reflect natural oceanographic inputs rather than anthropogenic pollution.

In contrast, the high Cd levels in *A. purpuratus* in Quintero–Puchuncaví are clearly linked to industrial sources. *A. purpuratus* is a well-known bioindicator of marine pollution due to its bioaccumulation capacity and commercial value [65]. Shellfish effectively accumulate Cd from their diets, making it difficult to eliminate from the body [66]. In Quintero-Puchuncaví, Cd concentrations in sediments have increased since 1967 and, unlike those in Quintay Bay, are linked to stationary and diffuse anthropogenic sources, including non-ferrous metal smelting, coal-fired power generation, and smelting slag [67]. Furthermore, Cr and Pb concentrations were elevated in the impacted area. Cr concentrations are typically higher near metallurgical and coal industry activities [68,69], and previously, high concentrations were detected in marine sediments from Quintero-Puchuncaví Bay, suggesting emissions from the coal-fired power plant [24]. Pb has also been detected in marine sediments associated with metal smelting industries [24,25]. Thus, their accumulation in benthic matrices is consistent with sediment-associated contamination pathways.

Hg concentrations in our study were similar in fish species across both areas. Similar results were found in the Bíobío region, southern Chile, including species such as *P. chilensis* and *Prolatilus jugularis*, even in sites that are not considered industrial hubs [70]. These levels were assumed to have originated from methylmercury (MeHg) accumulation, its organic form; although it is not the case in our study, high levels of MeHg can be accumulated additively in the food web and result in a risk to human health [71]. In our work, however, all Hg values were below the recommended threshold limits. Conversely, Cd concentrations exceeded or were close to the limits. Since metal pollution poses global risks to human health, guidelines have been established to set permissible levels of contaminants in seafood [72]. Crucially, a regulatory gap was identified; the Chilean decree does not currently set Cd limits for seafood or any animal meat, which may increase uncertainty about food safety for animal products. Moreover, no regulatory limits were found in Cd for gastropod species in any Chilean, EU [41] or CODEX [42] guidelines. Similarly, the Chilean decree maintains Pb thresholds (2.0 mg/kg) that are significantly higher than EU [41] and CODEX [42] standards (0.3 mg/kg). This investigation strongly supports a necessary revision and update of the Chilean Food Health Regulations to align with international standards and mitigate potential public health risks.

## 5. Conclusions

Quintero–Puchuncaví Bay represents a critical nexus of industrial activity and ecological risk where impacts on environmental and public health are well documented. Given the importance of artisanal fishing in this region, continuous monitoring of seafood contamination is essential. In this study, we assessed the concentrations of eight heavy metals in edible marine species, confirming systemic enrichment compared to the reference site at Quintay Bay. Our findings demonstrate that industrial pollution persists as a chronic stressor for local marine resources. While essential elements such as Zn and Cu exhibited high accumulation patterns, these levels likely reflect a synergy between physiological requirements and anthropogenic inputs. Cd stands out as a trend outlier, attributed to natural conditions likely associated with coastal upwelling. Additionally, the absence of regulatory thresholds for certain metals—and the fact that some limits in Chilean national standards are less stringent than international benchmarks—highlights the need to revise current regulations to ensure the protection of marine ecosystems and human health.

Notably, Cd concentrations exceeded international food safety thresholds in the fish species *P. chilensis* and *C. variegatus*, the mussel *A. purpuratus*, and the crabs *R. setosum* and *O. trimaculatus*, and THQ values were above 1 in *A. purpuratus* and *R. setosum*. Additionally, Pb concentrations at some sites exceeded or were near safety levels in the fish *A. punctatus* and again in *P. chilensis*. Given the high importance of these organisms in the local human diet, caution is strongly advised regarding their consumption; furthermore, authorities and decision-makers are advised to pay close attention to these issues for the management of current and future environmental and human health risks.

## Figures and Tables

**Table 1:** Mean ± standard deviation of metal concentrations (mg/kg dry weight) in the biota from the control and impacted areas of Quintero-Puchuncaví Bay.

Species	Area	n	Cd	Cr	Cu	Mn	Pb	V	Zn	Hg
*P. chilensis*	Quintay	7	0.05 ± 0.007	0.22 ± 0.04	1.16 ± 0.06	0.61 ± 0.18	0.28 ± 0.08	0.88 ± 0.05	12.51 ± 1.64	0.51 ± 0.009
	Q-P	4	0.18 ± 0.02	0.69 ± 0.14	1.56 ± 0.08	0.72 ± 0.27	0.50 ± 0.15	0.86 ± 0.05	14.68 ± 1.90	0.42 ± 0.004
*A. punctatus*	Quintay	4	0.04 ± 0.007	0.22 ± 0.04	1.11 ± 0.06	0.46 ± 0.16	0.36 ± 0.10	0.94 ± 0.06	20.25 ± 2.63	0.02 ± 2.2e-05
	Q-P	5	0.16 ± 0.02	0.66 ± 0.13	1.67 ± 0.08	0.61 ± 0.20	0.47 ± 0.14	0.87 ± 0.05	19.12 ± 2.50	0.42 ± 0.004
*C. variegatus*	Quintay	3	0.06 ± 0.006	0.2 ± 0.02	1.38 ± 0.05	0.36 ± 0.07	0.5 ± 0.09	0.86 ± 0.04	12.24 ± 0.98	0.5 ± 0.009
	Q-P	2	0.19 ± 0.03	0.60 ± 0.12	1.61 ± 0.08	0.37 ± 0.11	0.68 ± 0.2	0.77 ± 0.04	16.17 ± 2.10	0.48 ± 0.006
*C. concholepas*	Quintay	13	4.94 ± 0.74	0.17 ± 0.03	15.84 ± 0.88	1.47 ± 0.55	0.35 ± 0.10	3.13 ± 0.19	60.37 ± 7.84	0.02 ± 0.0004
	Q-P	4	5.71 ± 0.85	0.64 ± 0.10	58.61 ± 3.28	2.15 ± 0.81	0.61 ± 0.18	4.65 ± 0.27	228.6 ± 29.70	0.32 ± 0.001
*Fissurella* spp.	Quintay	12	0.97 ± 0.15	0.47 ± 0.09	11.92 ± 0.60	1.62 ± 0.49	0.86 ± 0.26	3.14 ± 0.19	19.88 ± 2.58	0.006 ± 0.0001
	Q-P	3	1.97 ± 0.30	2.81 ± 0.57	61.87 ± 3.09	4.79 ± 1.44	0.62 ± 0.19	4.34 ± 0.26	46.13 ± 5.99	0.02 ± 0.0003
*H. plana*	Quintay	5	0.56 ± 0.08	0.25 ± 0.05	46.93 ± 2.62	0.97 ± 0.36	0.38 ± 0.11	1.61 ± 0.09	225.2 ± 29.28	0.21 ± 0.005
	Q-P	8	0.77 ± 0.12	0.65 ± 0.13	59.43 ± 3.32	0.91 ± 0.34	0.58 ± 0.17	3.18 ± 0.19	240.40 ± 31.25	0.26 ± 0.003
*R. setosum*	Quintay	4	2.79 ± 0.41	0.39 ± 0.08	99.89 ± 5.59	1.92 ± 0.73	0.52 ± 0.15	2.69 ± 0.16	179.23 ± 23.30	0.08 ± 0.001
	Q-P	11	2.33 ± 0.36	0.72 ± 0.14	70.87 ± 3.96	0.99 ± 0.37	0.47 ± 0.14	2.51 ± 0.14	201.54 ± 26.20	0.25 ± 0.002
*L. trabeculata*	Quintay	2	3.12 ± 0.49	0.42 ± 0.09	1.28 ± 0.07	2.02 ± 0.78	0.58 ± 0.18	6.37 ± 0.39	8.17 ± 1.08	0.003 ± 0.0001
	Q-P	2	1.32 ± 0.20	3.53 ± 0.72	7.76 ± 0.43	7.20 ± 2.79	1.55 ± 0.47	6.54 ± 0.40	17.1 ± 2.25	0.02 ± 0.001
*A. purpuratus*	Q-P	6	11.08 ± 1.66	1.28 ± 0.26	30.85 ± 1.73	20.19 ± 7.82	1.99 ± 0.61	3.44 ± 0.21	112.1 ± 14.75	0.06 ± 0.001
*O. trimaculatus*	Q-P	6	3.64 ± 0.55	5.18 ± 1.06	108.73 ± 6.1	14.74 ± 5.71	1.05 ± 0.32	6.8 ± 0.42	189.99 ± 25	0.54 ± 0.007

## Data Availability

The original contributions presented in this study are included in the article/Supplementary Material. Further inquiries can be directed to the corresponding authors.

## Supplementary Materials

The following supporting information can be downloaded at: https://www.mdpi.com/article/10.3390/toxics14050397/s1, Table S1. Sampling sites along the Quintero-Puchuncaví Bay and Quintay Bay; Figure S1. Boxplot of metal concentrations (mg/kg) in *C. variegatus* from control and impacted areas; Figure S2. Boxplot of metal concentrations (mg/kg) in *Fissurella* spp. from control and impacted areas; Figure S3. Boxplot of metal concentrations (mg/kg) in *Lessonia trabeculata* from control and impacted areas; Figure S4. Principal Component Analysis (PCA) plot for the control (A) and impacted (B) areas. Table S2. Evaluation of metal contamination in fish, mollusk, and crustacean species (mg/kg w.w.) determined in our investigation and compared with other studies in Chile, also compared with different regulatory guidelines. Only results expressed in wet weight are included. Concentrations in red highlight levels meeting or above those proposed by food regulations; Table S3. THQ values for women and men in both Quintero-Puchuncaví Bay and Quintay Bay. Values in red highlight THQ levels ≥ 1 [73].
