# Supplementary material for: Heavy Metal Contamination in Edible Species from Quintero-Puchuncaví Bay: Risks Associated with the Icon Industrial Complex in Central Chile
Source: Toxics. 2026 May 6;14(5):397. doi: 10.3390/toxics14050397 (PMC13211075; doi:10.3390/toxics14050397)
Supplement: Supplementary file 1 [file toxics-14-00397-s001.zip › toxics-4243896-supplementary.pdf]

Supplementary material

Table S1. Sampling sites along the Quintero-Puchuncaví Bay and Quintay Bay

Site	Area	Latitude (°S)	Longitude (°W)
S1	Quintero-Puchuncaví	32°44′33″	71°29′56″
S2	Quintero-Puchuncaví	32°44′46″	71°29′48″
S3	Quintero-Puchuncaví	32°46′61″	71°30′66″
S4	Quintero-Puchuncaví	32°45′34″	71°29′21″
S5	Quintero-Puchuncaví	32°45′29″	71°29′38″
S6	Quintero-Puchuncaví	32°44′84″	71°29′45″
S7	Quintero-Puchuncaví	32°44′31″	71°29′22″
Control 1	Quintay	33°19'19"	71°42'58"
Control 2	Quintay	33°19'30"	71°41°30"

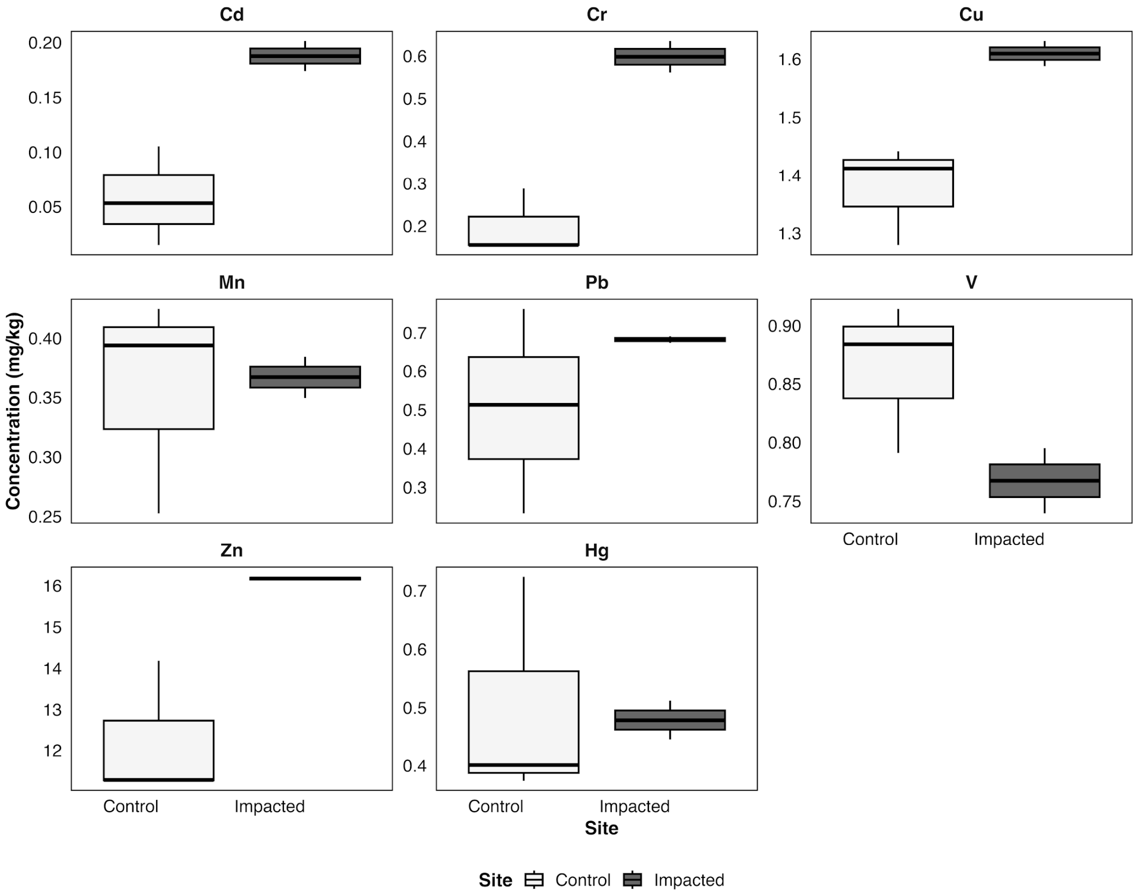

Figure S1. Boxplot of metal concentrations (mg/kg) in *C. variegatus* from control and impacted areas.

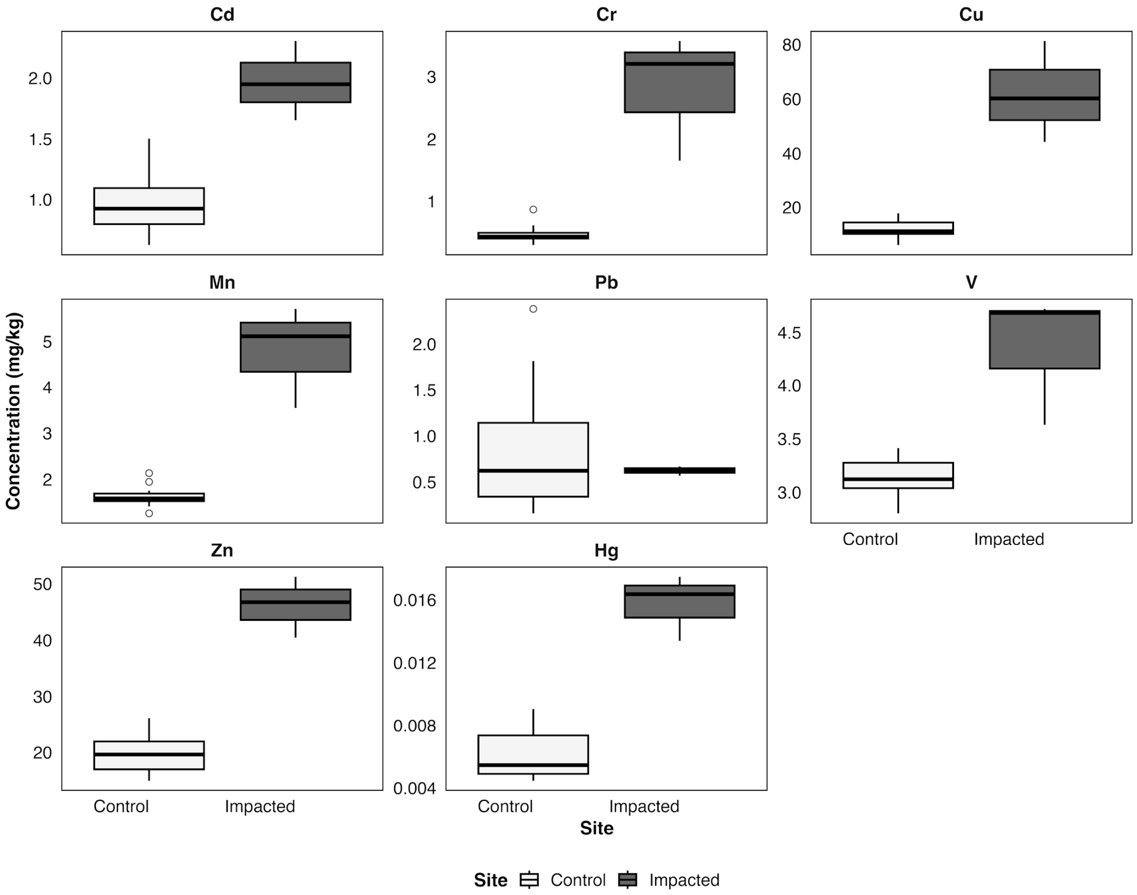

Figure S2. Boxplot of metal concentrations (mg/kg) in *Fissurella* spp. from control and impacted areas.

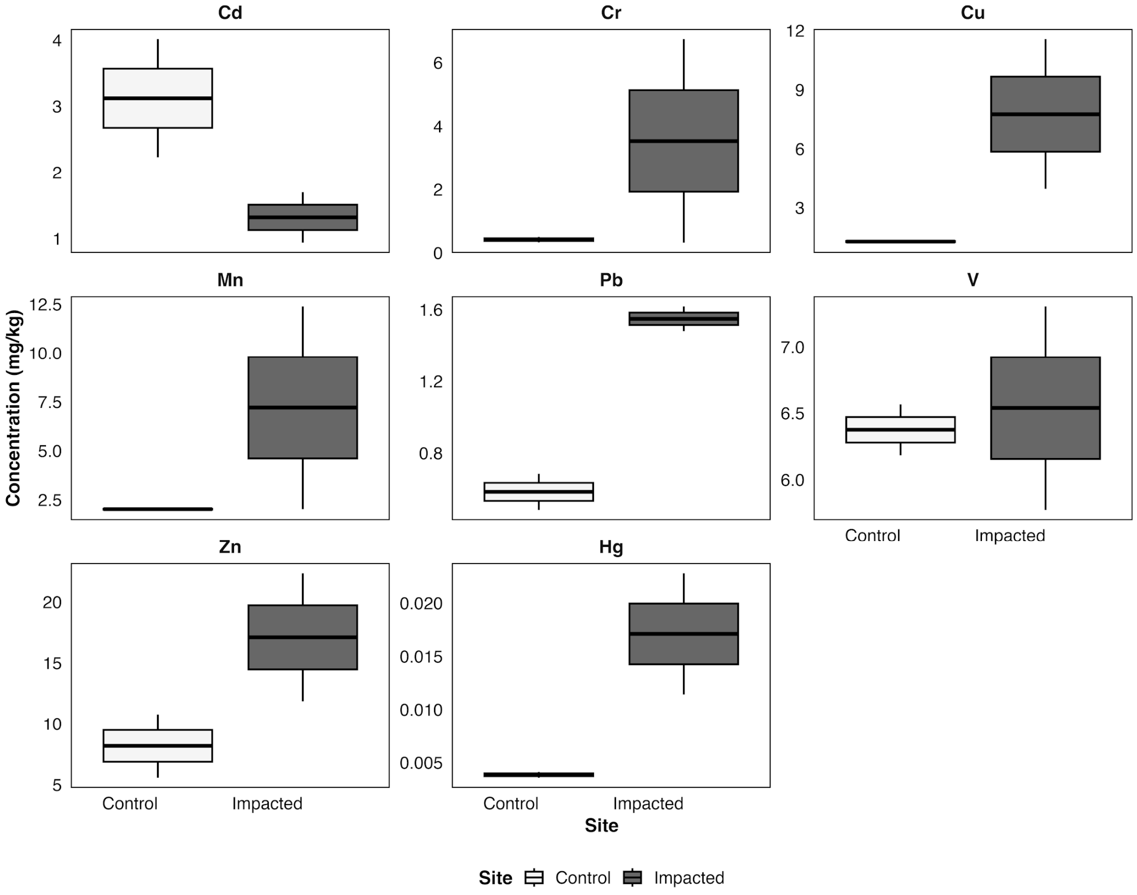

Figure S3. Boxplot of metal concentrations (mg/kg) in *Lessonia trabeculata* from control and impacted areas.

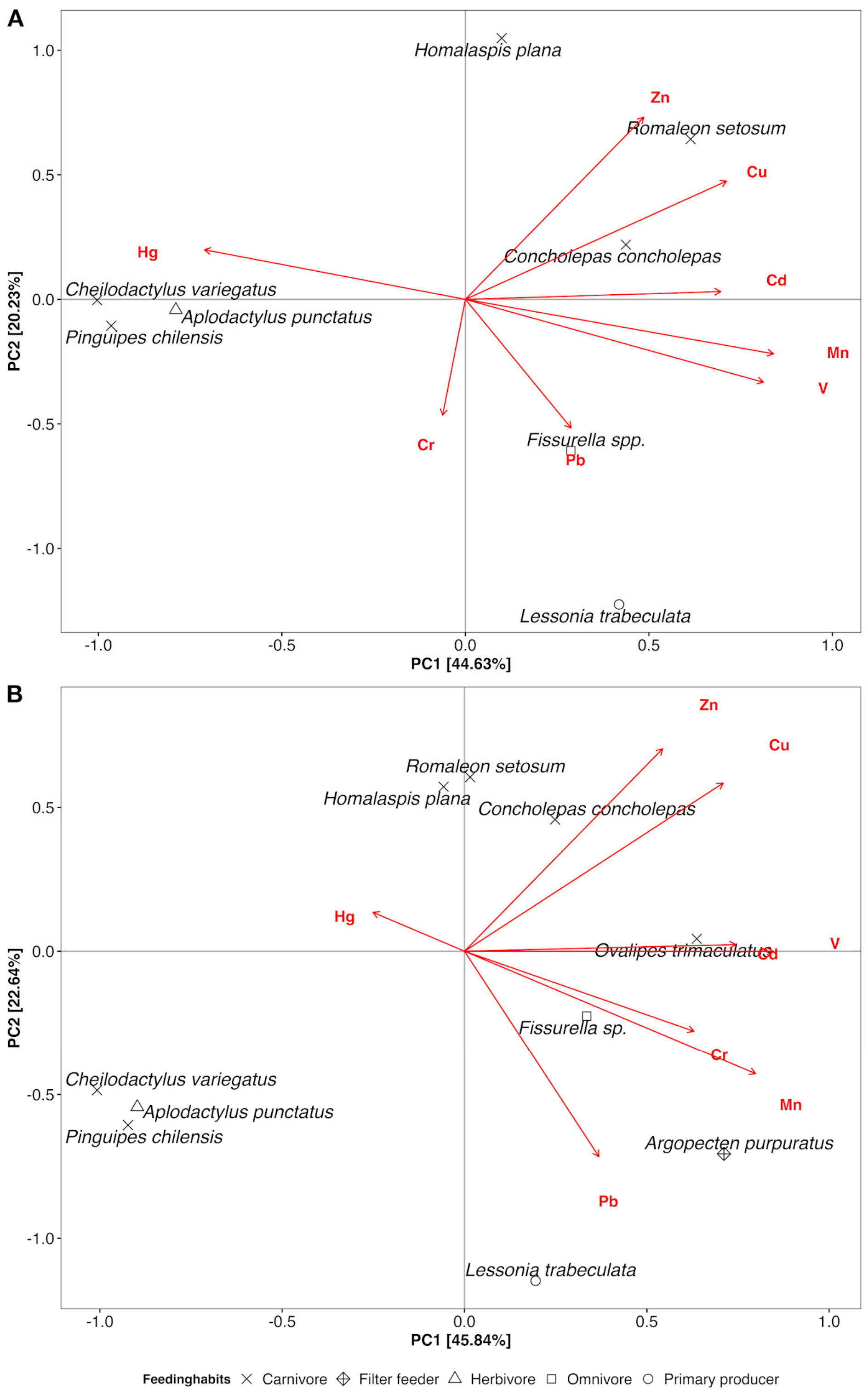

Figure S4. Principal Component analysis (PCA) plot for the control (A) and impacted (B) areas.

Table S2. Evaluation of metal contamination in fish, mollusk, and crustacean species (mg/kg w.w.) determined in our investigation and compared with other studies in Chile, also compared with different regulatory guidelines. Only results expressed in wet weight are included. Concentrations in red highlight levels meeting or above those proposed by food regulations.

Species	Metal	Concentration (mg/kg w.w)	Legal thresholds (mg/kg w.w)	Site	Reference
a) Values in this study					
P. chilensis	Cd	**0.05**	0.05[a]	Q-P	This study
		0.01	0.05[a]	Quintay	
	Pb	**0.22**	0.3[a,b], 2.0[c]	Q-P	
		0.07	0.3[a,b], 2.0[c]	Quintay	
	Hg	0.10	0.5[b,c]	Q-P	
		0.13	0.5[b,c]	Quintay	
A. punctatus	Cd	**0.04**	0.05[a]	Q-P	This study
		0.01	0.05[a]	Quintay	
	Pb	**0.28**	0.3[a,b], 2.0[c]	Q-P	
		0.09	0.3[a,b], 2.0[c]	Quintay	
	Hg	0.10	0.5[b,c]	Q-P	

		0.06	0.5[b,c]	Quintay	
C. variegatus	Cd	**0.05**	0.05[a]	Q-P	This study
		0.01	0.05[a]	Quintay	
	Pb	0.17	0.3[a,b], 2.0[c]	Q-P	
		0.13	0.3[a,b], 2.0[c]	Quintay	
	Hg	0.12	0.5[b,c]	Q-P	
		0.12	0.5[b,c]	Quintay	
C. concholepas	Cd	1.43	-	Q-P	This study
		1.9	-	Quintay	
	Pb	0.15	2.0[c]	Q-P	
		0.09	2.0[c]	Quintay	
	Hg	0.08	0.5[a,c]	Q-P	
		0.006	0.5[a,c]	Quintay	
Fissurella spp.	Cd	0.49	-	Q-P	This study
		0.24	-	Quintay	
	Pb	0.16	2.0[c]	Q-P	

		0.21	2.0[c]	Quintay	
	Hg	0.004	0.5[a,c]	Q-P	
		0.002	0.5[a,c]	Quintay	
R. setosum	Cd	**2.48**	0.5[a]	Q-P	This study
		**0.7**	0.5[a]	Quintay	
	Pb	0.12	0.5[a], 2.0[c]	Q-P	
		0.13	0.5[a], 2.0[c]	Quintay	
	Hg	0.06	0.5[a,c]	Q-P	
		0.02	0.5[a,c]	Quintay	
H. plana	Cd	0.19	0.5[a]	Q-P	This study
		0.14	0.5[a]	Quintay	
	Pb	0.14	0.5[a], 2.0[c]	Q-P	
		0.09	0.5[a], 2.0[c]	Quintay	
	Hg	0.07	0.5[a,c]	Q-P	
		0.05	0.5[a,c]	Quintay	
	Cd	**0.91**	0.5[a]	Q-P	This study

O. trimaculatus	Pb	0.26	0.5[a], 2.0[c]	Q-P	
	Hg	0.13	0.5[a,c]	Q-P	
A. purpuratus	Cd	**2.77**	1.0[a], 2.0[b]	Q-P	This study
	Pb	0.5	1.5[a], 2.0[c]	Q-P	
	Hg	0.01	0.5[a,c]	Q-P	
b) Concentrations in samples from different areas					
C. concholepas	Cd	16.2*	-	Cachagua	Castillo et al., 2023
		0.9*	-	Maitencillo	
		0.5*	-	Horcón	
		2.9*	-	Ventanas	
		0.7*	-	Farellones de Quintero	
		0.14*	-	Loncura	
		6.5*	-	Embarcadero	
		6.3*	-	NW Península de Los Molles	
		8.6*	-	Papagallo	
		0.01	-	Paraíso Beach	

		0.001	-	El Lenguado Beach	Castro and Valdés, 2012
	Pb	**27.9**	2.0[c]	La Chimba Beach	Valdés et al., 2014
		**53**	2.0[c]	Paraíso Beach	Castro and Valdés, 2012
		**21**	2.0[c]	El Lenguado Beach	
Fissurella spp.	Cd	0.004	-	El Lenguado Beach	Castro and Valdés, 2012
	Pb	**4.3**	2.0[c]	Carrizo Beach	Valdés et al., 2014
		**23.6**	2.0[c]	Puerto Beach	
		**13**	2.0[c]	El Lenguado Beach	Castro and Valdés, 2012
R. setosum	Cd	**2.4***	0.5[a]	Caldera Bay	Castillo and Valdés, 2011
		**10.9***	0.5[a]	Calderilla Bay	
		**4.7***	0.5[a]	Inglesa Bay	
		**30.4***	0.5[a]	Salada Bay	
	Pb	**60.2***	0.5[a], 2.0[c]	Caldera Bay	Castillo and Valdés, 2011
		**25.4***	0.5[a], 2.0[c]	Calderilla Bay	
		**80.5***	0.5[a], 2.0[c]	Inglesa Bay	

		**87.6***	0.5^a, 2.0^c	Salada Bay	
		**34.3**	0.5^a, 2.0^c	Coloso Beach	Valdés et al., 2014
A. purpuratus	Pb	**16.4**	1.5^a, 2.0^c	Coloso Beach	Valdés et al., 2014
		**16.2**	1.5^a, 2.0^c	La Chimba Beach	
		**36.2**	1.5^a, 2.0^c	La Rinconada Beach	

a. European Commission Regulation 2023/915. b. CODEX Alimentarius. c. Chilean Supreme Decree No. 977. (-) indicates limits not established. (*) Maximum concentration reported.

Table S3. THQ values for women and men in both Quintero–Puchuncaví Bay and Quintay Bay. Values in red highlight THQ levels ≥ 1.

Specie	Site	Cd		Pb		Hg	
		W	M	W	M	W	M
P. chilensis	Q-P	0.024	0.021	0.029	0.026	0.545	0.483
	Quintay	0.006	0.005	0.009	0.008	0.669	0.594
A. punctatus	Q-P	0.021	0.019	0.037	0.033	0.543	0.482
	Quintay	0.006	0.005	0.019	0.011	0.308	0.273
C. variegatus	Q-P	0.025	0.022	0.022	0.019	0.625	0.554
	Quintay	0.008	0.007	0.016	0.015	0.652	0.579
C.	Q-P	0.745	0.661	0.019	0.018	0.423	0.375

concholepas	Quintay	**0.990**	0.879	0.011	0.010	0.032	0.029
Fissurella spp.	Q-P	0.257	0.228	0.020	0.018	0.021	0.018
	Quintay	0.126	0.112	0.028	0.025	0.008	0.007
R. setosum	Q-P	**1.297**	**1.151**	0.015	0.014	0.323	0.287
	Quintay	0.365	0.323	0.017	0.015	0.101	0.089
H. plana	Q-P	0.100	0.089	0.019	0.017	0.345	0.306
	Quintay	0.073	0.065	0.012	0.011	0.269	0.239
O. trimaculatus	Q-P	0.476	0.422	0.034	0.030	0.704	0.624
A. purpuratus	Q-P	**1.448**	**1.284**	0.065	0.058	0.076	0.068

References

Castillo, A., and Valdés, J. (2011). Contenido de metales en *Cancer polyodon* (Crustacea: Decapoda) en un sistema de bahías del norte de Chile (27°S). Latin American Journal of Aquatic Research, 39(3), 461–470. https://doi.org/10.3856/vol39-issue3-fulltext-7

Castillo, A.; Valdés, J.; Marambio, Y.; Figueroa, L.; Letelier, J.; Carcamo, F. Metal(loid)s content in *Concholepas concholepas* (Mollusca) and human health assessment in a coastal environmental sacrifice zone, central Chile (~32°S). Mar. Pollut. Bull., 2023, 197, 115738. https://doi.org/10.1016/j.marpolbul.2023.115738

Castro, G., and Valdés, J. (2012). Concentración de metales pesados (Cu, Ni, Zn, Cd, Pb) en la biota y sedimentos de una playa artificial, en la bahía San Jorge 23°S, norte de Chile. Latin

American Journal of Aquatic Research, 40(2), 267–281. https://doi.org/10.3856/vol40-issue2-fulltext-3

Valdés, J., Guiñez, M., Castillo, A., and Vega, S. E. (2014). Cu, Pb, and Zn content in sediments and benthic organisms from San Jorge Bay (northern Chile): Accumulation and biotransference in subtidal coastal systems. Ciencias Marinas, 40(1), 45–58. https://doi.org/10.7773/cm.v40i1.2318
